# BPV associated with imaging features of SSI on MRI

**DOI:** 10.1002/brb3.2155

**Published:** 2021-05-07

**Authors:** Yong Peng Yu, Ya Li Zheng, Lan Tan, Ting Ting Jiang

**Affiliations:** ^1^ Department of Neurology Weihai Central Hospital Affiliated to Qingdao University Weihai China; ^2^ Department of Neurology Weihai Central Hospital Affiliated to Weifang Medical College Weihai China; ^3^ Department of Neurology Qingdao Municipal Hospital Affiliated to Qingdao University Qingdao China

**Keywords:** blood pressure variability, day‐to‐day, lacunar infarction, visit‐to‐visit

## Abstract

**Objectives:**

A retrospective study was performed to investigate the relationship between blood pressure variability (BPV) and imaging features of single small infarction (SSI) on magnetic resonance imaging (MRI).

**Materials and Methods:**

Two hundreds and five patients with SSI and 120 healthy subjects matched with age and sex as the control group were enrolled into this study. All subjects came from the Affiliated Hospital to Qingdao University and Qingdao Municipal Hospital from October 2011 to June 2016. Research subjects were classified into different groups. Blood pressure was measured once a day and recorded during the hospitalization period (7–10 days). The followed up data of patients after discharging from hospital was collected from the follow‐up records.

**Results:**

Twenty‐four hours BPV (SBP_Mean_, DSBP_Max_, DSBP_SD_, NDBP_Max_, NDBP_SD,_ and DDBP_CV_), day‐to‐day, and visit‐to‐visit BPV (SBP_Max_, SBP_SD_, DBP_Max,_ and DBP_SD_) in the SSI group were significantly higher than that in control group. Compared with the giant lacunar group, day‐to‐day BPV (SBP_Mean_, SBP_Max_, SBP_SD_, SBP_CV_, DBP_Mean_, DBP_Max_, DBP_SD_), and visit‐to‐visit BPV (SBP_Mean_, SBP_Max_, SBP_SD_, DBP_Mean_, DBP_Max_, DBP_SD_) were significantly higher in the small lacunar infarct group (*p* < .05). The 24 hr BPV (SBP_Mean_, DDBP_Max_, DDBP_Mean_), day‐to‐day BPV (SBP_Max_, SBP_SD_, SBP_CV_), and visit‐to‐visit SBP_Max_ in nonround lesion group were significantly higher than that in round group (*p* < .05). Compared with nondeep lesion group, some parameters in day‐to‐day BPV and visit‐to‐visit BPV were significantly higher in the deep small lesion group (*p* < .05).

**Conclusion:**

Increased BPV parameters such as day‐to‐day and visit‐to‐visit (SBP_Max_, SBP_SD_, DBP_Max_) were related to the SSI characterized by small lesion in deep brain region.

## Graphical abstract

A retrospective study was performed to investigate the relationship between blood pressure variability (BPV) and imaging features of single small infarction (SSI) on magnetic resonance imaging (MRI). Increased BPV parameters such as day‐to‐day and visit‐to‐visit (SBPMax, SBPSD, and DBPMax) were related to the SSI characterized by small in deep brain region.

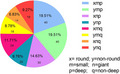

## 1 INTRODUCTION

Hypertension is the most vital risk factor for a variety of vascular events (Lawes et al., 2008), but independent of mean blood pressure, blood pressure variability (BPV) has been associated with clinical cardiovascular and cerebrovascular diseases. Blood pressure variability (BPV) can be classified into very short‐term, short‐term, mid‐term, and long‐term BPV. Long‐term BPV can lead to subclinical organ damage, poor renal function, all‐cause mortality, and also cardiovascular events (Soh et al., 2019). Previous studies had shown that stroke‐related target organ damage is closely connected with BPV (Takase et al., 2011). Independent stroke prognosis information carried by BPV will change our current understanding of the importance of blood pressure (Veluw et al., 2017). Clinical studies have confirmed that improving BPV is another important goal in managing blood pressure level (Turtzo et al., 2009). There is a lot of evidence that the predictive effect of BPV on target organ damage is independent of the mean blood pressure. The visit‐to‐visit systolic BPV and maximal systolic blood pressure (SBP) are predictors of stroke independent of the mean systolic blood pressure. Rothwell et al. (2010a) found that the maximum of visit‐to‐visit systolic blood pressure and systolic BPV are independent risk factors for cardiovascular and cerebrovascular events (Rothwell et al. 2010a; Rothwell et al. 2010b). The predictive value of visit‐to‐visit BPV for cardiovascular events is higher than average blood pressure and 24 hr BPV. Lacunar infarction was always found both in well‐controlled hypertensive patients and in nonhypertensive subjects (Musini & Wright, 2009). Hypertensive factors are currently considered to play a dominant role in the development of small lacunar infarction.

Although the visit‐to‐visit BPV is linked to the incidence of stroke, the degree of association between visit‐to‐visit BPV and stroke may vary from different study populations. It has been noted that elevated SBP is a risk factor for lacunar infarction (Chen et al., 2008). While few studies have involved in the relationship between BPV and imaging feature of SSI on MRI. Therefore, this study was performed to investigate the relationship between BPV and imaging feature of SSI on MRI.

## 2 MATERIALS AND METHODS

### 2.1 Patients

All subjects who were enrolled into this study came from the Affiliated Hospital to Qingdao University and Qingdao Municipal Hospital from October 2011 to June 2016. Two hundred and five patients with SSI were enrolled into this study according to inclusion criteria and exclusion criteria. ECG, echocardiography, intracranial and extracranial MRA (or CTA), TCD, and carotid color ultrasonography were used to search for etiologies. The etiological classification was based on the Chinese Ischemic Stroke Subtype (CISS) classification standard (Gao et al., 2011). Depending on the purpose of the study, different grouping methods were used: the patients were divided into round‐shaped lesions and nonround lesions (wedge, triangle, and irregular) according to the morphology of the lesions; giant lacunar lesions group (40 mm > d ≥ 20 mm) and small lacunar lesions group (d < 20 mm) according to lesion size; deep infarction group (lesion located in basal ganglia, internal capsule, thalamus, brain stem); and nondeep infarction group (focus in cortex, radiography crown, and semi‐ovoid center) according to lesion distribution. At the same time, in order to investigate the relationship between BPV and SSI, one hundred and twenty healthy subjects matched with age and gender were collected as the control group.

*Inclusion criteria*: (1) All cases were confirmed as new infarction (DWI positive and low apparent diffusion coefficient) by cranial MRI; (2) Hospitalized within 3 days after onset, the diameter of cerebral infarction was less than 40 mm in DWI. *Exclusion criteria*: (1) With obvious stroke sequelae; (2) Large areas of cerebral infarction (complete posterior circulation infarction and over 1/3 anterior circulation); (3) Transient ischemic attack (TIA); (4) Combined intracranial hemorrhage and hemorrhagic transformation after cerebral infarction, infection, and tumor; (5) With severe heart, liver, kidney, and other underlying diseases; (6) Age > 80 years, or with severe dementia or Parkinson's disease and Parkinson's syndrome; (7) Nervous system demyelinating diseases such as Guillain–Barre syndrome, multiple sclerosis, etc.; (8) Disturbance of consciousness. The hospital's institutional review committee on human research approved this study protocol.

### 2.2 Clinical and imaging assessment

The severity of neurological impairments of the index stroke was measured using the National Institutes of Health Stroke Scale (NIHSS). NIHSS scores were recorded when patients were admitted to hospital. The risk factors including gender, smoking, alcoholism, hypertension, coronary heart disease, diabetes, fasting blood glucose, and high blood lipids were recorded. The main risk factors were determined as follow: (1) hypertension: antihypertensive drugs before admission or systolic blood pressure > 140 mmHg, or diastolic blood pressure ≥ 90 mmHg after hospital admission; (2) type 2 diabetes: a history of diabetes; fasting blood glucose >7.0 mmol/L or blood glucose 2 hr after meal > 11.1 mmol/L, while glycated hemoglobin >6.5%; (3) hyperlipidemia: past dyslipidemia or abnormalities after hospital admission, total cholesterol > 5.72 mmol/L, TG > 1.72 mmol/L, or LDL > 3.12 mmol/L; (4) Smoking: Currently smoking or quitting smoking (10 sticks/day for 5 years or more (5) Drinking: Daily alcohol consumption > 50ml, abstinence or not drinking).

The cranial MRI scan was performed within 72 hr after admission, and GE's superconducting magnetic resonance imaging equipment (model: GE MR Discovery 750 3.0T) was used to obtain T1WI, T2WI, and DWI, 3D‐TOF MRA, and SWI images. The scanning parameters were set as follow: FLAIR‐T1WI (TR/TE, 1750ms/24 ms, TI 780ms; FOV 24 cm×24cm, matrix 320 × 256); FRFSE‐T2WI (TR/TE, 4300 ms/95ms, FOV 24 cm × 24 cm, matrix number of 512 × 512); DWI‐EPI (TR/TE, 3000 ms/70 ms; FOV 24 cm × 24 cm, matrix 160 × 160. The patients in the hospital would complete CTA or MRA within one week. Artery stenosis was divided into normal, mild stenosis (<50%), or moderate to severe stenosis (≥50%). The lesions of acute cerebral infarction show high signal on DWI, low signal on ADC, and hyperintensity in the FLAIR image. The maximum diameter of the lesion shown by DWI was regarded as the diameter of the lesion. Diameter (d) = 20 mm was regarded as the critical point. Lesion with d < 20 mm is regarded as a small lacuna. For DWI being very sensitive to small lesions, and there is no lower limitation for the lesion diameter. Lesion with 40 mm > d ≥ 20 mm is regarded as giant lacuna. SSI was determined as follow: lesions occur in the cortex, cortex infarcts in the lower half of the oval center and in the coronal, basal ganglia, inner cystic striatum, thalamus, and brainstem regions, regardless of whether the main artery has any degree of stenosis, not considering the size of the lesion (not to exceed 1/3 anterior circulation, the maximum of transverse diameter of the lesion not exceed 40 mm). Neurologists and neuro‐radiologists integrated the clinical manifestations and neuroimaging findings, respectively.

### 2.3 The followed up data collecting about 24 hr ambulatory blood pressure and follow‐up blood pressure

All the followed up data collected from the follow‐up records were analyzed. Twenty‐four‐hour ambulatory blood pressure measurement: in order to minimize the adverse disturbance of hyperacute cerebral infarction on blood pressure, the MC‐6800 ambulatory blood pressure monitoring (Shenzhen Meili Biomedical Electronics Co., Ltd.,) was performed after the onset of the disease once every 60 min, continuously monitor the blood pressure for 24 hr.08:00 and 20:00 were set as two time periods of blood pressure monitoring during the day and night. For some patients with acute cerebral infarction who were treated with intravenous thrombolysis after admission, ambulatory blood pressure monitoring was performed 24 hr after admission. The effective SBP measurement ranges from 70 mmHg to 260 mmHg, and DBP 50 mmHg to 150 mmHg. 24 hr effective blood pressure reading >90% is qualified. During hospitalization (general hospitalization 7–10 days), blood pressure is measured and recorded every morning. Patients were followed up after discharge. Patients were referred once a month in the outpatient clinic, and blood pressure measurements were taken at rest for 5 min and recorded. Parameters of blood pressure included as follow: daytime maximum systolic blood pressure (DSBP_Max_), daytime maximum diastolic pressure (DDBP_Max_), nighttime maximum systolic blood pressure (NSBP_Max_), nighttime maximum diastolic blood pressure (NDBP_Max_), 24 hr systolic blood pressure, 24 hr SBP_Mean_), 24 hr mean diastolic blood pressure (24 hr DBP_Mean_), daytime systolic blood pressure (DSBP_Mean_), daytime diastolic blood pressure (DDBP_Mean_), and mean nighttime systolic blood pressure (night systolic blood pressure, NSBP_Mean_), nighttime diastolic blood pressure (NDBP_Mean_), daytime systolic blood pressure coefficient of variation (DSBP_CV_), nighttime systolic blood pressure (nighttime systolic blood pressure)‐coefficient of variation (NSBP_CV_), daytime diastolic blood pressure coefficient of variation (DDBP_CV_), nighttime diastolic blood pressure coefficient of variation (NDBP_CV_), standard deviation of daytime systolic blood pressure (daytime systolic blood pressure‐standard deviation (DSBP_SD_), nighttime systolic blood pressure‐standard deviation (NSBP_SD_), day diastolic blood pressure‐standard deviation (DDBP_SD_), and standard deviation of night diastolic blood pressure (nighttime diastolic blood pressure‐standard deviation, NDBP_SD_). The SBP_Mean_, SBP, DBP, SD, CV, and other parameters were collected. The CV calculation method is CV = 100 × *SD*/mean.

### 2.4 Statistical analysis

Visit‐to‐visit BPV was quantified by calculating the maximum (Max), standard deviation (SD), coefficient of variation (100 × SD/mean). Measurement data and count data were expressed as mean ± standard deviation (Mean ± SD) and percentage. The *t*‐test was used for data with normal distribution of data. The Mann–Whitney *U* test was used for nonparametric data that did not meet the normal distribution. The chi‐square test was used for the count data. A logistic regression analysis was thus performed, before and after adjustment, for the effect of the potential confounding variables to evaluate the risk relationship between the radiographic phenotypes of SSI and BPV parameters. The following potential confounders were considered: demographic characteristics (sex, age), baseline MMSE score, vascular risk factors (hypertension, diabetes, smoking habits, and hyperlipidemia).SPSS 16.0 package for Windows was used for statistical analysis. *p* < .05 was considered statistically significant.

## 3 RESULTS

### 3.1 Demographic and clinical characteristics of participants

A total of 205 patients with SSI were enrolled into this study. The average age was (69.4 ± 10.1) years, of which 113 (55.1%) were male. The average age of the control group including 120 healthy subjects was (68.1 ± 10.8) years, of which 64 (53.3%) were male. The baseline characteristics of the SSI and noncerebral infarction groups are shown in Table 1. There were statistically significant differences in risk factors such as blood lipids, blood glucose, and hypertension (*p* < .05) between them. There were nonround cerebral infarction in 97 cases (36.5%), round‐like cerebral infarction in 108 cases (63.5%). 122 cases of small lacuna lesions and 83 cases of giant lacunar lesions. According to the distribution of infarction lesions, patients were divided into deep infarction group (130 cases) (lesions located in the basal ganglia, internal capsule, thalamus, brainstem), and nondeep infarction group (75 cases) (lesions located in the cortex, radiant crown, and semi‐oval center). Baseline characteristics of every subgroup showed in the Table 2.

**TABLE 1:** Demographic and clinical characteristics of patients in the infarcts and noninfarct groups

Variables	Infarcts group (*n* = 205)	Noninfarct group (*n* = 120)	*p* value
Age [years, *M* (SD)]	69.4 ± 10.1	68.1 ± 10.8	.16
Men (*n*, %)	113 (55.1%)	66 (55.0%)	.48
Diabetes mellitus (*n*, %)	27 (13.2%)	14 (11.7%)	.67
Hypertension (*n*, %)	78 (38.3%)	36 (30.0%)	.14
Hyperlipidemia (*n*, %)	35 (17.1%)	10 (8.3%)	.027
Coronary heart disease (*n*, %)	13 (6.3%)	5 (4.2%)	.40
Atrial fibrillation (*n*, %)	9 (4.4%)	6 (5.0%)	.011
History of stroke (*n*, %)	16 (7.8%)	5 (4.1%)	<.01
Smoking (*n*, %)	26 (12.7%)	10 (8.3%)	.24
Alcohol (*n*, %)	36 (17.6%)	17 (14.2%)	.42
TCH [mmol/L, *M* (*SD*)]	5.10 (0.83)	4.68 (0.87)	<.001
TG [mmol/L, *M* (*SD*)]	1.09 (0.29)	1.01 (0.23)	<.01
LDL [mmol/L, *M* (*SD*)]	3.34 (0.75)	3.15 (0.58)	<.01
HLDL [mmol/L, *M* (*SD*)]	1.83 (0.27)	1.73 (0.31)	<.01
Glu [mmol/L, *M* (*SD*)]	7.36 (3.01)	6.4 (2.5)	<.01

**TABLE 2:** Clinical baseline characteristics of subgroups of SSI

Variables	Round (*n* = 108)	Nonround (*n* = 97)	*p*	Small lacunar (*n* = 122)	Giant lacunar (*n* = 83)	*p*	Deep infarction (*n* = 130)	Nondeep infarction (*n* = 75)	*p*
Age [years, *M* (*SD*)]	70.1 (9.1)	68.7 (11.1)	.16	70.2 (10.3)	68.4 (9.9)	.10	69.2 (9.6)	69.7 (10.9)	.36
Men (*n*, %)	63 (58.3%)	50 (51.5%)	.08	72 (59.5%)	41 (48.8%)	.12	67 (54.4%)	46 (56.1%)	.41
Diabetes mellitus (*n*, %)	14 (12.9%)	13 (13.4%)	.93	8 (6.1%)	19 (22.6%)	<.01	14 (11.4%)	13 (15.8%)	.35
Hypertension (*n*, %)	42 (38.8%)	36 (37.1%)	.70	41 (33.9%)	37 (44.0%)	.032	45 (36.6%)	33 (40.2%)	.60
Hyperlipidemia (*n*, %)	20 (18.5%)	15 (15.5%)	.57	20 (15.3%)	15 (17.8%)	.80	24 (19.5%)	11 (13.4%)	.25
CHD (*n*, %)	7 (6.5%)	6 (6.2%)	.95	6 (4.9%)	7 (8.3%)	.56	11 (8.9%)	2 (2.4%)	.06
Atrial fibrillation (*n*, %)	4 (3.7%)	5 (5.1%)	.80	6 (4.6%)	3 (3.6%)	.81	7 (5.7%)	2 (2.4%)	.44
History of stroke (*n*, %)	8 (7.4%)	8 (8.2%)	.88	6 (4.9%)	10 (12.0%)	.20	10 (8.1%)	6 (7.3%)	.03
Smoking (*n*, %)	12 (11.1%)	14 (14.4%)	.54	15 (12.4%)	11 (13.2%)	.93	12 (9.7%)	14 (17.1%)	.12
Alcohol (*n*, %)	16 (14.8%)	20 (18.1%)	.28	25 (19.1%)	11 (13.1%)	.16	18 (14.6%)	18 (21.9%)	.18
TCH [mmol/L, *M* (*SD*)]	5.14 (0.82)	5.05 (0.84)	<.01	5.11 (0.84)	5.09 (0.83)	.43	5.16 (0.87)	5.01 (0.77)	.10
TG [mmol/L, *M* (*SD*)]	1.09 (0.27)	1.08 (0.31)	.40	1.08 (0.30)	1.09 (0.26)	.40	1.09 (0.26)	1.08 (0.33)	.40
LDL [mmol/L, *M* (*SD*)]	3.33 (0.77)	3.36 (0.73)	.38	3.27 (0.76)	3.39 (0.70)	.12	3.31 (0.78)	3.36 (0.71)	.42
HLDL[mmol/L, *M* (*SD*)]	1.82 (0.28)	1.84 (0.27)	.31	1.82 (0.25)	1.85 (0.30)	.21	1.83 (0.27)	1.84 (0.26)	.40
Glu [mmol/L, *M* (*SD*)]	7.25 (3.10)	7.50 (2.95)	.27	7.32 (3.14)	7.42 (2.86)	.40	7.08 (2.90)	7.78 (3.16)	.05
NIHSS [score, *M* (*SD*)]	2.77 (1.60)	2.4 (1.54)	.04	2.0 (1.38)	3.42 (1.48)	<.01	2.63 (1.62)	2.54 (1.51)	.34
Small lesion (*n*, %)	70 (64.8%)	42 (43.2%)	<.01				76 (61.7%)	46 (56.1%)	.10
Deep infarction (*n*, %)	81 (75.0%)	42 (43.3%)	<.01	75 (61.4%)	48 (57.6%)	.20			

### 3.2 The composition of the different imaging feature categories of SSI

**FIGURE 1:**
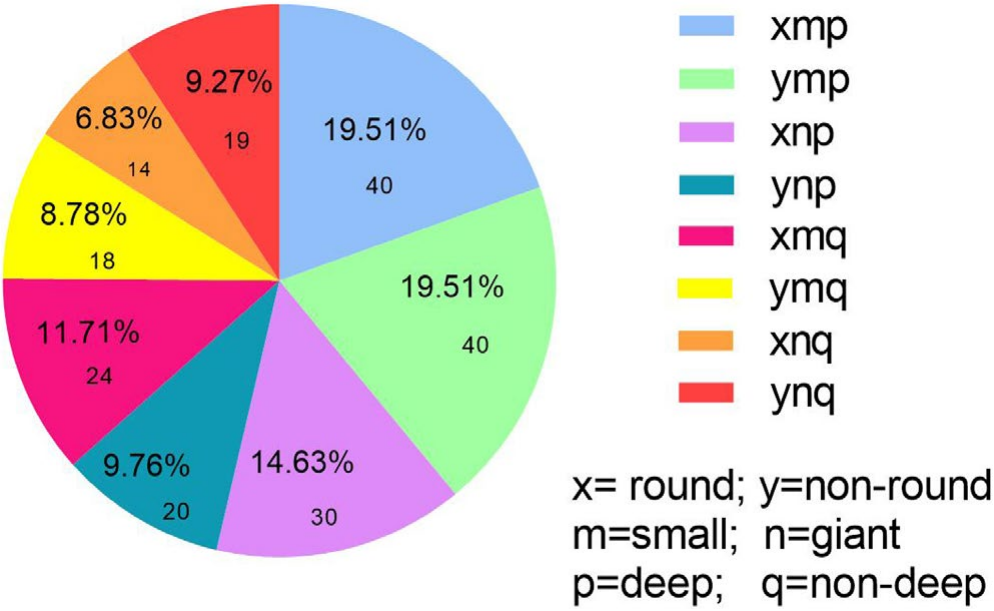
The composition of the different imaging feature categories of SSI including *xmp, ymp*, *xnp*, *ynp, xmq*, *ymp*, *xnp*, and *ynp* models. *x*, round lesion; *y*, nonround lesion; *m*, small lacunar lesion; *n*, giant lacunar lesion; *p*, deep lesion; *q*, nondeep lesion

In the present study, MRI imaging features of SSI lesion were defined as *x *= round lesion (round), *y *= nonround lesion (nonround), *m* = small lacunar lesion (small), *n *= giant lacunar lesion (giant), *p *= deep lesion (deep), *q* = nondeep lesion (nondeep). Figure 1 shows the composition of the different imaging feature categories of SSI including *xmp* model (40 cases, 19.51%), *ymp* model (40 cases, 19.51%), *xnp* model (30 cases, 14.63%), *ynp* model (20 cases, 9.76%), *xmq* model (24 cases, 11.71%), *ymp* model (18 cases, 8.78%), *xnp* model (14 cases, 6.83%), *ynp* model (19 cases, 9.27%). Round lesions and deep lesions account for 52.68% and 63.41% in SSI lesions, respectively.

### 3.3 The correlation between BPV and SSI

Compared with the noninfarction group, the SBP_Mean_, DSBP_Max_, DSBP_SD_, NDBP_Max_, NDBP_SD,_ and DDBP_CV_ in the SSI group were significantly higher in the aspect of 24 hr BPV. There was a statistically significant difference between these two groups (*p* < .05, Table 3). SBP_Max_, SBP_SD_, DBP_Max_, and DBP_SD_ were significantly higher in the SSI group, and the difference between these two groups was statistically significant in the aspect of day‐to‐day and visit‐to‐visit BPV (*p* < .05). Visit‐to‐visit SBP_CV_ was higher in the SSI group that in the noninfarction group. There was no statistically significant difference between these two groups (*p* = .06, *p* = .07), but the clinical significance should not be ignored (Table 3).

**TABLE 3:** Comparison of BPV parameters of 24 hr, day‐to‐day and visit‐to‐visit among different subgroups of SSI

Variables	Infarct group (*n* = 205)	Noninfarct group (*n* = 120)	*p* value
24 hr BPV			
SBP_Mean_	144.9 ± 12.5	144.5 ± 11.8	.102
DSBP			
Max	164.1 ± 18.8	152.0 ± 12.8	<.001
SD	12.7 ± 4.0	9.8 ± 4.3	<.001
CV	8.4 ± 2.9	8.0 ± 3.4	.08
NSBP			
Max	158.3 ± 15.6	148.9 ± 12.9	<.001
SD	10.9 ± 4.2	8.4 ± 3.8	<.01
CV	7.5 ± 2.8	6.9 ± 3.8	.35
DBP_Mean_	81.4 ± 3.1	79.5 ± 8.0	.04
DDBP			
Max	93.5 ± 2.7	89.5 ± 4.2	.08
SD	8.3 ± 2.7	7.3 ± 2.9	<.001
CV	10.3 ± 3.4	9.4 ± 3.1	.01
NDBP			
Max	88.0 ± 8.1	83.7 ± 7.7	<.001
SD	6.3 ± 2.0	5.5 ± 1.8	<.001
CV	7.5 ± 2.5	7.1 ± 2.5	.24
Day‐to‐day BPV			
SBP_Mean_	142.8 ± 10.8	136.9 ± 11.8	<.001
Max	152.1 ± 14.9	146.7 ± 11.0	<.001
SD	11.4 ± 5.4	7.9 ± 4.3	<.001
CV	8.3 ± 3.8	7.8 ± 3.5	.11
DBP_Mean_	83.5 ± 6.4	82.15 ± 5.8	.10
Max	93.2 ± 7.9	88.6 ± 6.1	<.001
SD	8.0 ± 2.5	6.1 ± 2.8	<.001
CV	9.7 ± 3.4	7.9 ± 3.7	.002
Visit‐to visit BPV			
SBP_Mean_	143.5 ± 10.7	141.8 ± 9.8	.06
Max	153.7 ± 12.1	148.3 ± 10.6	<.001
SD	9.7 ± 4.5	7.5 ± 4.0	<.001
CV	11.8 ± 5.0	8.1 ± 3.9	.07
DBP_Mean_	83.1 ± 7.4	82.5 ± 6.7	.23
Max	93.8 ± 7.3	91.2 ± 6.5	<.001
SD	7.8 ± 2.4	7.1 ± 2.3	.014
CV	9.4 ± 3.0	9.1 ± 2.8	.12

Abbreviations: BPV, blood pressure variability.

### 3.4 Comparison of BPV parameters of 24 hr, day‐to‐day and visit‐to‐visit in different subgroups of SSI

In the aspect of 24 hr BPV parameters, SBP_Mean_, DDBP_Max,_ and DDBP_Mean_ were significantly different between the round group and the nonround group. SBP_Mean_, DDBP_Max_ were higher in the round group, whereas DDBP_Mean_ was higher in the nonround group. There was no statistically significant trend in 24 hr BPV parameters between the small lacunar lesions group and giant lacunar lesions group. There was no significant difference in 24 hr BPV between deep infarction and nondeep infarction (*p* > .05).

In terms of day‐to‐day BPV parameters, SBP_Max_, SBP_SD,_ and SBP_CV_ were statistically significant in the round group and the nonround group (*p* < .05), and were higher in the nonround group. SBP_Mean_, SBP_Max_, SBP_SD_, SBP_CV_, DBP_Mean_, DBP_Max,_ and DBP_SD_ were statistically significant between the small lacunar lesions group and giant lacunar lesions group. (*p* < .05), and the above indicators were higher in the small lacunar lesions group. Compared with nondeep infarction group, SBP_Mean_, SBP_Max_, DBP_Mean,_ and DBP_Max_ in deep infarction group were significantly increased, and there was statistically significant between these two groups (*p* < .05). There were statistically significant differences in SBP_Max_ and DBP_Max_ among these four subgroups. The other parameters were not statistically different among these subgroups (*p* > .05).

In terms of visit‐to‐visit BPV parameters, SBP_Max_ was statistically significant between the round group and the nonround group (*p* < .05), and was higher in the nonround group. SBP_Mean_, SBP_Max_, SBP_SD_, DBP_Mean_, DBP_Max,_ and DBP_SD_ were statistically significant between the small lacunar lesions group and giant lacunar lesions group (*p* < .05). Compared with nondeep infarction group, SBP_Mean_ and SBP_Max_ were significantly increased in the deep infarction group (*p* < .05). (Table 4).

**TABLE 4:** Comparison of BPV parameters of 24 hr, day‐to‐day and visit‐to‐visit in different subgroups of SSI

Variables	Round lesion (*n* = 108)	Nonround lesion (*n* = 97)	*p*	Small lacunar lesion (*n* = 122)	Giant lacunar lesion (*n* = 83)	*p*	Deep infarction (*n* = 130)	Nondeep infarction (*n* = 75)	*p*
24 hr									
SBP_Mean_	147.6 (12.1)	142.9 (12.7)	<.01	145.3 (11.9)	145.5 (13.5)	.45	146.3 (12.1)	144.0 (13.1)	.097
DSBP									
Max	163.9 (19.3)	164.5 (18.2)	.41	164.6 (17.3)	163.7 (20.7)	.36	165.2 (18.2)	162.7 (19.5)	.175
SD	12.9 (4.2)	12.6 (3.5)	.15	12.2 (4.0)	12.3 (3.94)	.42	12.0 (3.8)	12.6 (4.2)	.119
CV	8.4 (2.8)	8.5 (3.1)	.40	8.4 (2.8)	8.6 (3.1)	.31	8.2 (2.7)	8.9 (3.2)	.060
NSBP									
Max	158.2 (15.8)	158.4 (15.3)	.46	158.4 (14.9)	158.1 (16.5)	.44	159.4 (15.3)	156.6 (15.8)	.098
SD	10.8 (4.5)	11.2 (3.5)	.24	10.4 (3.6)	11.5 (4.5)	.43	10.6 (4.0)	11.4 (4.0)	.075
CV	7.3 (3.1)	7.8 (2.5)	.10	7.0 (2.5)	8.0 (3.3)	.045	7.1 (2.7)	8.0 (2.9)	.039
DBP_Mean_	80.9 (3.3)	82.1 (2.8)	<.01	81.6 (3.7)	81.2 (3.0)	.36	81.5 (3.6)	81.3 (3.8)	.441
DDBP									
Max	94.0 (2.8)	93.1 (2.8)	.011	93.5 (3.1)	93.7 (2.5.5)	.43	94.0 (3.4)	92.8 (2.4)	.139
SD	8.2 (2.5)	8.5 (2.8)	.12	8.6 (2.8)	8.1 (2.5)	.09	8.6 (2.8)	7.9 (2.4)	.042
CV	10.2 (3.3)	10.4 (3.6)	.33	10.8 (3.7)	9.7 (3.2)	.50	10.6 (3.5)	9.9 (3.2)	.065
NDBP									
Max	88.4 (8.2)	87.6 (7.9)	.24	88.3 (8.3)	87.6 (7.8)	.28	88.2 (7.9)	87.7 (8.3)	.356
SD	6.2 (1.9)	6.5 (2.2)	.15	6.4 (2.2)	6.3 (1.9)	.36	6.3 (2.1)	6.4 (1.9)	.327
CV	7.3 (2.2)	7.8 (2.8)	.07	7.8 (2.7)	7.3 (2.2)	.08	7.6 (2.6)	7.5 (2.3)	.426
Day‐to‐day									
SBP_Mean_	142.1 (11.2)	143.6 (10.3)	.074	145.9 (11.1)	140.6 (10.0)	.022	144.4 (11.0)	141.2 (10.3)	.018
Max	151.3 (14.5)	153.0 (15.2)	.046	155.8 (15.1)	148.4 (14.4)	<.01	154.6 (14.6)	150.0 (15.0)	.016
SD	10.8 (4.6)	12.9 (6.0)	.003	12.7 (5.7)	10.5 (4.9)	.005	12.1 (5.2)	10.8 (5.6)	.153
CV	7.6 (3.4)	9.2 (4.2)	.007	8.8 (4.1)	7.5 (3.4)	.010	8.4 (3.7)	8.0 (4.0)	.247
DBP									
Mean	83.1 (7.0)	83.7 (6.2)	.243	85.1 (7.0)	80.9 (5.3)	<.01	84.2 (7.0)	82.1 (5.9)	.016
Max	92.4 (7.2)	94.2 (8.5)	.049	95.5 (8.2)	90.0 (6.1)	<.01	94.2 (8.1)	91.8 (7.3)	.015
SD	8.0 (2.4)	8.1 (2.6)	.373	8.5 (2.7)	7.5 (2.6)	.009	8.1 (2.8)	7.8 (2.6)	.219
CV	9.7 (3.6)	9.7 (3.2)	.478	10.0 (3.4)	9.3 (3.4)	.092	9.7 (3.5)	9.6 (3.3)	.389
Visit‐to‐visit									
SBP_Mean_	142.7 (11.3)	144.5 (10.0)	.113	145.1 (10.7)	141.3 (10.4)	.006	144.8 (10.7)	143.0 (10.6)	.009
Max	152.5 (12.2)	155.1 (12.1)	.047	157.6 (11.2)	149.4 (9.3)	<.01	155.9 (11.5)	150.4 (9.4)	<.001
SD	9.8 (4.3)	9.7 (4.30)	.429	9.9 (4.3)	9.4 (3.9)	<.01	9.85 (4.3)	9.6 (3.9)	.186
CV	11.9 (5.3)	11.7 (4.7)	.481	11.9 (5.1)	11.8 (4.9)	.305	12.3 (5.2)	11.4 (4.8)	.194
DBP									
Mean	83.0 (7.0)	83.2 (7.9)	.430	85.0 (7.5)	80.3 (6.5)	<.01	83.1 (7.5)	83.1 (7.4)	.246
Max	94.3 (6.8)	93.2 (8.1)	.152	94.8 (7.6)	92.5 (6.5)	.013	94.2 (7.4)	93.47 (7.0)	.104
SD	7.9 (2.6)	7.7 (2.3)	.224	8.1 (2.4)	7.1 (2.7)	.060	7.8 (2.4)	7.8 (2.6)	.233
CV	9.6 (3.1)	9.3 (2.9)	.237	9.6 (2.8)	9.2 (3.2)	.180	9.4 (2.9)	9.6 (3.2)	.15

### 3.5 Logistic regression analysis for the relation between BPV and imaging feature of SSI on MRI

After adjusting the confounding factors, some BPV parameters were independently associated with SSI. The SSI lesions in the deep brain territory were most closely related to the increase of BPV which includes the three parameters of day‐to‐day and visit‐to‐visit SBP_Max_, SBP_SD,_ and DBP_Max_. (Table 5).

**TABLE 5:** Independent risk factors analysis for different classification of SSI and BPV

	Variables	Day‐to‐day BPV			Visit‐to‐visit BPV		
		*OR*	95%CI	*p* value	*OR*	95%CI	*p* value
Small l lesion	SBP_Max_	1.8	1.33–2.81	.040	1.32	1.03–1.138	<.01
	SBP_SD_	1.10	1.015–1.33	.025	1.24	1.31–1.46	.022
	DBP_Max_	1.13	1.08–1.26	.028	2.58	1.69–4.20	<.01
Non‐ round lesion	SBP_Max_	1.04	1.0–1.08	.052	1.11	1.02–1.32	.043
	SBP_SD_	1.18	1.35–1.98	.036	–	–	–
Deep lesion	SBP_Max_	1.02	1.003–1.05	.038	1.151	1.25–1.69	<.01
	SBP_SD_	1.14	1.005–1.24	.035	1.20	1.08–1.36	<.01
	DBP_Max_	1.12	1.032–1.30	.041	1.19	1.30–1.72	<.01

## 4 DISCUSSION

### 4.1 BPV correlates with SSI

SSI accounts for about 60%–70% of cerebral infarction in clinical practice. Although BPV is closely related to the development of lacunar infarction, brain white matter hyperintensity (WMH), CMBs and enlarged perivascular space, the correlation between different evaluation indexes of BPV with the related diseases are not completely consistent. A number of studies had shown that 24 hr BPV was associated with high white matter signal, and the correlation between BPV and high severity and WMH at different location was not exactly the same (Tartaro et al., 1999; Brickman et al., 2010). Because short‐term BPV was affected by temporary external objective conditions, it might interfere with or obscure the intrinsic link between BPV and disease. The short‐term and long‐term BPV contain different information about the changes in blood pressure, which have different prediction effects on disease. The correlation between SSI lesions including the cortical and its subcortical areas, basal ganglia, thalamus, internal capsule, and brainstem with short‐term and long‐term BPV still remains elusive. This present study found that, in the aspect of 24 hr short‐term BPV, there were statistically significant in the day‐to‐day DSBP_Max_, DSBP_SD_ and NDBP_Max_ and NDBP_SD_. The day‐to‐day BPV (SBP_Max_, SBP_SD_, DBP_Max_, DBP_SD,_ and DBP_CV_) and visit‐to‐visit BPV (SBP_Mean_, SBP_Max_, SBP_SD_, DBP_Max,_ and DBP_SD_) in SSI patients increased significantly compared with that in the noncerebral infarction group. Previous studies had shown that BPV was associated with the recurrence of lacunar infarction (Yamaguchi et al., 2014; Lee et al., 2014). The results of this study further provide the relation between different categories of BPV with imaging feature of SSI on MRI.BPV parameters closely related to SSI mainly focus on the maximum and standard deviations of systolic and diastolic blood pressure.

### 4.2 BPV related to imaging feature of SSI on MRI

In the present study, 64.8% of lacunar infarction appeared as round‐shaped lesions. This form of infarction can be caused by the opening and terminal lesion in the single perforator artery. The results showed that 61.4% of the SSI lesions were located in the deep brain. Seventy‐five percent of round lesions belonged to deep brain infarction, suggesting that different imaging feature of SSI had different propensity distribution. Small and round lacunar lesions are tending to locate in deep brain area such as basal ganglia and thalamus. The proportion of hypertension in the small lacunar lesion group was higher than that in the giant lacunar lesion group, but there was not statistically significant in the 24‐hr ambulatory blood pressure parameters between these two groups. However, there was a significant increase in visit‐to‐visit BPV in the small lacunar lesion group. The maximum of SBP was significantly increased in the non‐round lesion group and the small lacunar lesion group. Hypertension, SBP and DBP have similar epidemiological features in both regular and irregular lacunar infarction, whereas BPV is more pronounced in irregular lesions (Feng et al., 2013). The results of this study suggest that long‐term BPV is closely related to nonround lesions. The imaging features of small focal in deep brain structures were closely related to BPV parameters of day‐to‐day and visit‐to‐visit including SBP_Max_, SBP_SD,_ and DBP_Max_. This study found that systolic and diastolic blood pressure peaks and systolic BPV were independent high‐risk factors for deep focal infarction. Hypertension mainly results in hyaline degeneration or fibrinoid necrosis of arterioles, consequently resulting in lacunar infarction or microinfarction. Clinical and animal experimental studies have demonstrated that long‐term hypertension with significant BPV can lead to severe small‐vascular endothelial dysfunction (Diaz et al., 2012), leading to rupture of the blood–brain barrier and expansion of the Virchow–Robin spaces (VRS) (Klarenbeek et al., 2013).

Increased visit‐to‐visit BPV will promote the progression of small cerebral vascular disease (Liu et al., 2012). The abnormal biological effects of BPV may be the key underlying mechanism for the relationship between hypertension and small infarcts found in many previous researches. In 2017, AHA/ACC the Hypertension Clinical Practice Guide which reduced the standard for the diagnosis of hypertension by 10 mmHg. At present, the hypertension guidelines of all countries use the blood pressure or blood pressure average value in the consulting room as the evaluation index for the diagnosis and treatment of hypertension, ignoring the influence of BPV on the target organs and the prognosis of cardiovascular and cerebrovascular diseases. Recent studies have shown that BPV, a measure of blood pressure stability, is closely related to the risk of cardiovascular and cerebrovascular events. This study found that the distribution frequency of SSI lesions from high to low was basal ganglia, brainstem, centrum semiovale and corona radiata, thalamus, and internal capsule, which was similar to previous studies (Arboix & Martí‐Vilalta, 2009; Beckmann et al., 2010). It is known that lacunar infarction is most closely related to hypertension (Altmann et al., 2015; Das et al., 2008), and lacunar infarction is mostly belong to SSI in the deep brain. Patients with SSI in deep brain had higher BPV parameters; especially visit‐to‐visit BPV was significantly higher in patients with SSI in deep brain than that with SSI patients in nondeep brain. Different pattern of BPV might be related to different distribution patterns of SSI. The incidence of infarcted lesions was higher in the basal ganglia, brainstem, and centrum semiovale and corona radiata. BPV was associated with impaired endothelial function in small vessels (Diaz et al., 2012). The BPV factor has greater influence on the change of tensile stress that the blood vessel wall is underwent. However, there was no significant difference in 24 hr ambulatory blood pressure parameters between these two groups.

There are several limitations in this study. Firstly, although we have adjusted many covariates, there may be residual confounding which potentially influence this result. In addition, the judgment of SSI is based on the first MRI scan after admission. Patients with cerebral infarction who did not underwent re‐examination of the cranial MRI. Given that the imaging feature of the SSI can change over time. Therefore, small infarct lesions were initially diagnosed as small lacunar, which may become giant lacunar with the time going. With the expansion of lesions, round lesions may become nonround lesions. Thirdly, the study was performed based on the Chinese patients, so it would be not generalizable to other races or ethics. Further study should be performed to confirm the optimal time point for day‐to‐day and visit‐to‐visit BPV reduction in SSI patients. Whether long‐term BPV reduction can be converted to long‐term clinical benefit for preventing SSI still remains uncertain. A large sample of prospective randomized controlled clinical study is still needed.

## 5 CONCLUSION

This study finds that increased BPV parameters such as day‐to‐day and visit‐to‐visit (SBP_Max_, SBP_SD_, DBP_Max_) were related to the SSI characterized by small lesion in deep brain region. Of course, this conclusion still needs to be confirmed by prospective studies. In addition to 24 hr average blood pressure, long‐term BPV, blood pressure peak, maximum control of rapid fluctuations should be focus on.

## CONFLICT OF INTEREST

None declared.

## AUTHOR CONTRIBUTIONS

Putting forward this idea and project administration: Yong peng Yu. Data curation, Methodology: Yong peng Yu, Lan Tan, Yali Zheng and Tingting Jiang. Software and Writing, review and editing: Yong peng Yu and Yali Zheng.

### PEER REVIEW

The peer review history for this article is available at https://publons.com/publon/10.1002/brb3.2155.

## Data Availability

The data that support the findings of this study are available from the corresponding author upon reasonable request.
